# Supplementary material for: Overexpression Populus d-Type Cyclin Gene PsnCYCD1;1 Influences Cell Division and Produces Curved Leaf in Arabidopsis thaliana
Source: Int J Mol Sci. 2021 May 29;22(11):5837. doi: 10.3390/ijms22115837 (PMC8197873; doi:10.3390/ijms22115837)
Supplement: Supplementary file 1 [file ijms-22-05837-s001.zip › ijms-1231007-supplementary.pdf]

Supplementary Files

Supplementary Table

Supplementary Table 1 Primer sequences used in this study

Name	Sequences
PsnCYCD1;1-flag-*Xba* I-F	5’- ATCTCTAGAGAGATGGCCTACTCTGATTGCTT ATCAG -3’
PsnCYCD1;1-flag-*Sac* I-R	5’- ATCGAGCTCCTAAGCCTTGTCATCGTC ATCCTTGTAGTCGCTCTCGGAATTTCCTTTGTCATC-3’
PsnCYCD1;1-RT-F	5’- CCTCTGGTTCCTTCTCTATTGG -3’
PsnCYCD1;1-RT-R	5’- CAGAAACCCAGTATAGGCTCC -3’
Atactin-RT-F	5’- TGCCAATCTACGAGGGTTTC-3’
Atactin-RT-R	5’- TCTCTTACAATTTCCCGCTCTG -3’
Atactin-RT-F	5’- AGAGATTCAGATGCCCAGAAGTCTTGTTCC -3’
Atactin-RT-R	5’- AACGATTCCTGGACCTGCCTCATCATACTC -3’
AtE2F1-RT-F	5’- ACCTACCATGCTTCCAGAATAAG -3’
AtE2F1-RT-R	5’- CATCAGGGACATTAGAGGGTTC -3’
AtE2F2-RT-F	5’- TGAGGAAAGCAGGTTGGATG -3’
AtE2F2-RT-R	5’- GCCCCATTCTACTCCTAATCAC -3’
AtE2F3-RT-F	5’- GCAGAGTCAACATCAAGTCAAAG -3’
AtE2F3-RT-R	5’- AGGTCCAGCATTCCATCTTTG -3’
AtELP1-RT-F	5’- AGCGGGTATTTGGAACAGAG -3’
AtELP1-RT-R	5’- ACCTTCCCTTTCCGACTTTG -3’
AtELP2-RT-F	5’- ACTTGCCGTTACTTCTCCTTC -3’
AtELP2-RT-R	5’- AAAACCCCAACACTCTCCAG -3’
AtELP3-RT-F	5’- CTGTTCCTCGTGCTCTATCTG -3’
AtELP3-RT-R	5’- GGGAGATGGTGAGAACTCTTG -3’
AtDPB-RT-F	5’- ACGCAACAGTAGAAGTGGAG -3’
AtDPB-RT-R	5’- CTTTGTTAGGGTTTTCTGGCG -3’
AtDPA-RT-F	5’- AGATGGTTGTGAAGACTCAAGG -3’
AtDPA-RT-R	5’- GCTATGTTGAGATTGGTGATGTG -3’
AtRb-RT-F	5’- TCTGCCAAAACCTTCTCCTG -3’
AtRb-RT-R	5’- GCTTCTGCTTTACACATTGCC -3’
AtH4-RT-F	5’- AGGAAAAGGGTTAGGCAAAGG -3’
AtH4-RT-R	5’- AACATCCATAGCAGTCACCG -3’
AtEXP10-RT-F	5’- TGGACTTAGCTGTGGTTCTTG -3’
AtEXP10-RT-R	5’- GCAAGGTCAAAGTGTTCAAGAG -3’
AtSTM-RT-F	5’- CCAACAGGATGTCTAGGTGAAG -3’
AtSTM-RT-R	5’- GAGAAAGAGGAAGGTGAGGATAG -3’
AtKNAT1-RT-F	5’- ACAAGAGAATAACAGCGGAGG -3’
AtKNAT1-RT-R	5’- CAACGCTACCTTCTCTGACTC -3’
AtKNAT2-RT-F	5’- ACCGGAGACAATCAAAGACTG -3’

AtKNAT2-RT-R	5'- GTAGGTTTGGAGTAAGCGAGG -3'
AtAS1-RT-F	5'- CGGTTGTGATGGCTAATTCG -3'
AtAS1-RT-R	5'- ACTGTGGAAGGCGATAATGTC -3'
AtAS2-RT-F	5'- GCGAAAATGTCAACCGGAATG -3'
AtAS2-RT-R	5'- TGACGAAGCTGATGTTGGAG -3'
AtPHB-RT-F	5'- CAGATGTCGTGAGAAGCAGAG -3'
AtPHB-RT-R	5'- CTGAGGATTTGGGTTTTGCTG -3'
AtPHV-RT-F	5'- GAGAAGCAGAGGAAAGAGTCAG -3'
AtPHV-RT-R	5'- CTGATGTGTTGGGTTTTGCTG -3'
AtANT-RT-F	5'- AACAAAACCAAAACCAAAACCAC -3'
AtANT-RT-R	5'- GAGCTTCATATCTACCAGTCCATC -3'

Supplementary Figure

Supplementary Fig. 1 Homology analysis of PsnCYCD1;1 amino acid

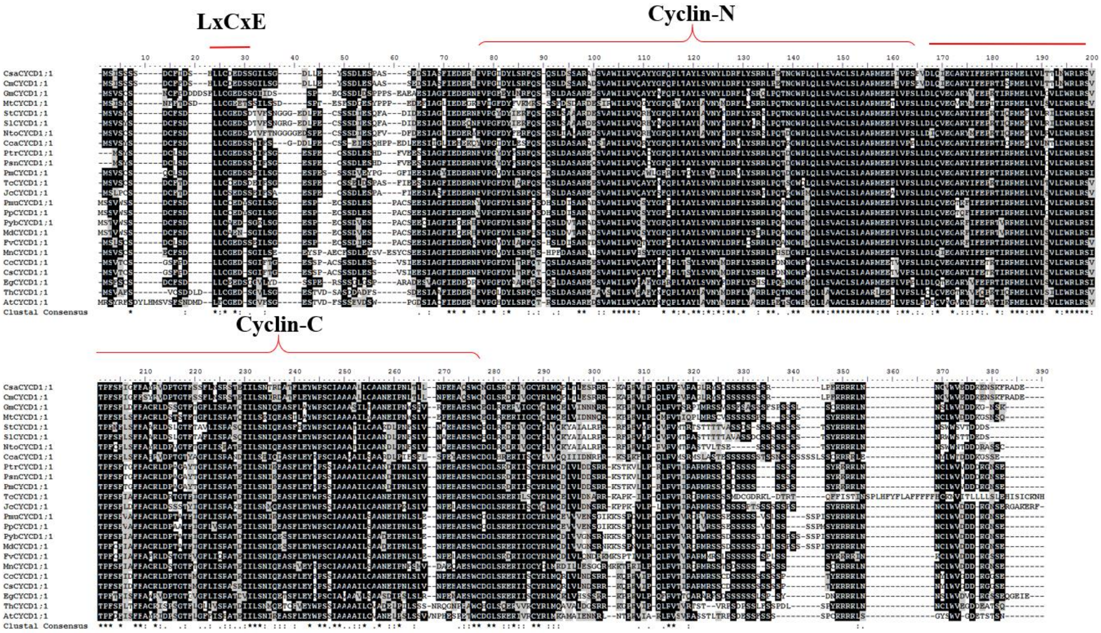

Supplementary Fig. 2 Genetic transformation and identification of PsnCYCD1;1-flag in *A. thaliana.* **A**. Resistance screening of *Arabidopsis* seedlings. **B**. The growth of resistant seedlings under short-day condition. **C**. The growth of resistant seedlings under long-day condition. **D**. PCR detection of 13 *Arabidopsis* resistant seedlings. M, DNA marker DL 5000; P, pROKII-*PsnCYCD1;1-flag* vector as positive control; WT, wild type; 1-13, twelve PCR products of Arabidopsis resistant seedlings; 14. negative control.

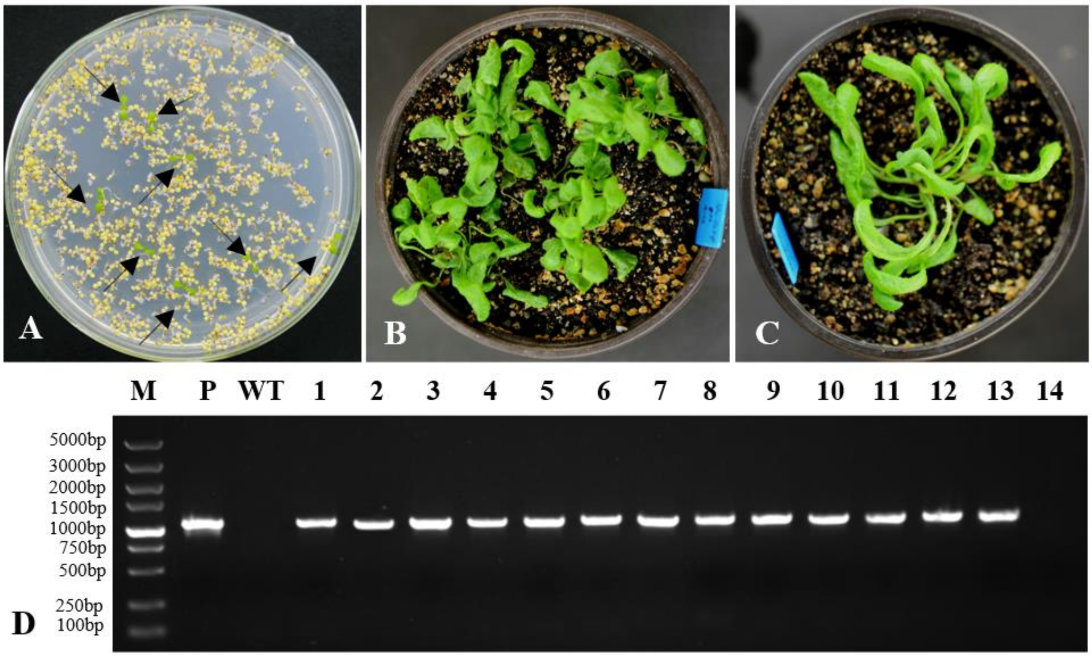

Supplementary Fig. 3 Phenotypic analysis of transgenic *Arabidopsis* seedlings. **A-C**. Wild type *Arabidopsis* seedlings. **D-F**. Type I transgenic *Arabidopsis* seedlings. **G-I**. Type II transgenic *Arabidopsis* seedlings. **J**. Comparison of hypocotyl length between wild-type and transgenic *Arabidopsis* seedlings. WT, wild type *Arabidopsis* seedlings; Type I, Type I *Arabidopsis* seedlings; Type II, Type II *Arabidopsis* seedlings.

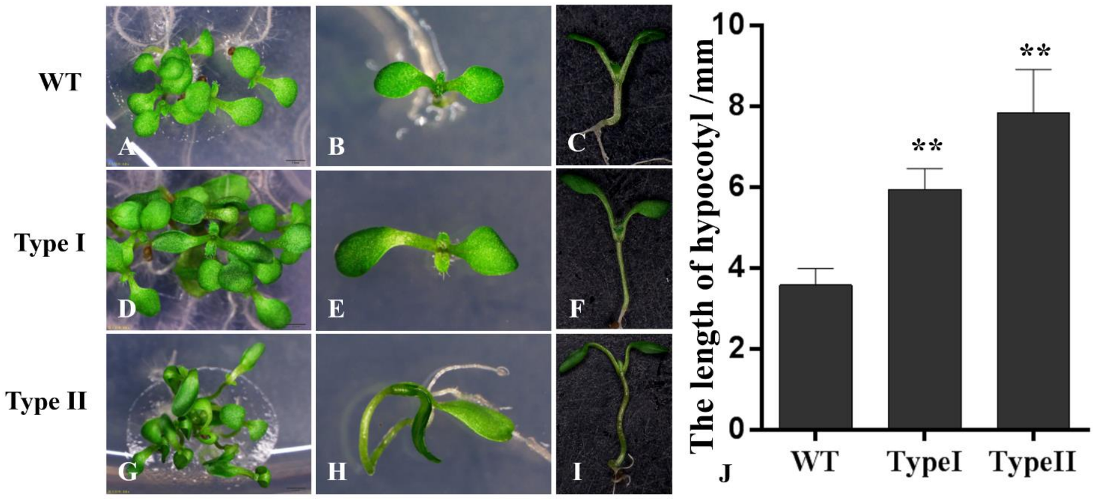

Supplementary Fig. 4 Comparative analysis of root length of transgenic *Arabidopsis* seedlings. **A**, **B**. Vertical cultivation of wild type and transgenic *Arabidopsis* roots. **C**. Statistical analysis of the root length of wild type and transgenic *Arabidopsis* seedlings. WT, wild type *Arabidopsis* seedlings; Type I, type I *Arabidopsis* seedlings; Type II, type II *Arabidopsis* seedlings.

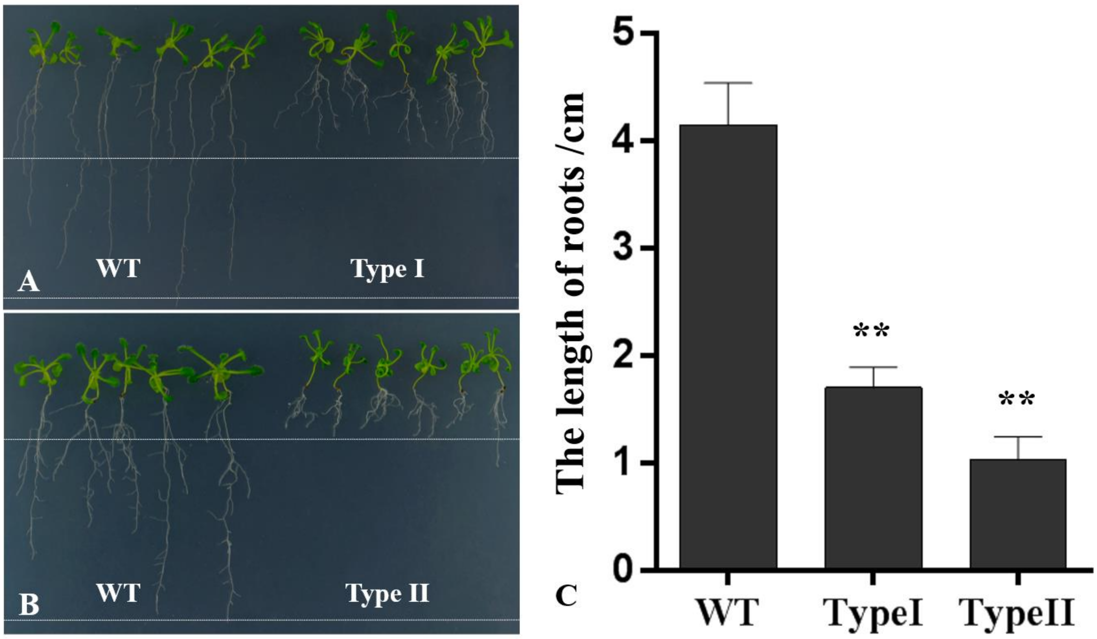
A
WT
Type I
B
WT
Type II
C
The length of roots /cm
5
4
3
2
1
0
**
**
WT
TypeI
TypeII
